# In Situ Characterization Method to Reveal the Surface Reconstruction Process of an Electrocatalyst

**DOI:** 10.3390/nano15120917

**Published:** 2025-06-12

**Authors:** Yiqin Zhan, Tao Yang, Shuang Liu, Liming Yang, Enhui Wang, Xiangtao Yu, Hongyang Wang, Kuo-Chih Chou, Xinmei Hou

**Affiliations:** 1 State Key Laboratory of Environmental Criteria and Risk Assessment, Chinese Research Academy of Environmental Sciences, Beijing 100012, China; 2 Institute for Carbon Neutrality, University of Science and Technology Beijing, Beijing 100083, China; 3 Institute of Steel Sustainable Technology, Liaoning Academy of Materials, Shenyang 110000, China; 4 Beijing Advanced Innovation Center for Materials Genome Engineering, University of Science and Technology Beijing, Beijing 100083, China

**Keywords:** electrolysis of water, surface reconstruction, electrocatalyst, in situ characterization

## Abstract

Renewable energy-driven water electrolysis is widely regarded as a pivotal approach for achieving carbon-free hydrogen production. The development of highly efficient electrocatalysts is crucial to advancing the efficiency and scalability of electrolytic water splitting. Recent advancements in characterization techniques have revealed that catalysts often undergo surface reconstruction during the hydrogen evolution reaction (HER) and oxygen evolution reaction (OER), leading to the formation of real active species. Understanding the surface reconstruction process through advanced characterization methods is essential for the rational design of high-performance catalysts. However, the surface reconstruction of catalysts is a highly complex phenomenon, and conventional ex situ characterization techniques often fall short of capturing the dynamic evolution of the catalyst surface. Consequently, in situ characterization methods have emerged as indispensable tools for elucidating the surface reconstruction process. This paper provides a detailed review of the process of surface reconstruction, the reasons behind it, and the in situ characterization methods, and finally discusses the challenges faced by the characterization methods for the reconstruction of water electrolysis catalysts in future development.

## 1. Introduction

The use of traditional fossil fuels accounts for more than 80% of global carbon emissions and is central to achieving carbon neutrality. Amidst a transformative transition in the worldwide energy paradigm, characterized by a gradual departure from reliance on fossil fuels, hydrogen energy has surfaced as a viable contender in the realm of sustainable energy alternatives. Due to its versatility and potential for zero-carbon emissions, hydrogen is increasingly recognized as a key enabler of the clean energy transition. Among the diverse array of hydrogen production methodologies, pathways powered by renewable energy sources, especially water electrolysis, have attracted considerable scientific and industrial interest. This approach offers a sustainable and environmentally friendly alternative, as it entirely eliminates emissions of SO_x_, NO_x_, CO_2_, and CO, thereby addressing critical environmental challenges associated with conventional energy production [1].

The electrocatalytic water-splitting hydrogen production system comprises several essential components, including an electrolyzer, a membrane, an electrolyte, an anode catalyst for the OER, a cathode catalyst for the HER, a power supply, and a gas–liquid separator. Among these, the catalyst system plays a pivotal role, as it directly determines both hydrogen production efficiency and overall energy conversion performance. The advancement of efficient electrocatalytic systems plays a pivotal role in optimizing hydrogen production efficacy, minimizing energy expenditure, and maintaining sustained operational reliability. Both acidic and alkaline electrolysis systems operate under extreme pH conditions, elevated temperatures, and highly corrosive environments. These harsh operational conditions inevitably induce surface reconstruction of electrocatalysts during HER and OER, significantly impacting their catalytic activity and long-term stability. Therefore, a comprehensive grasp and meticulous regulation of the surface restructuring mechanisms of catalysts during electrolysis are imperative for the strategic development of robust and high-performance electrocatalytic materials.

During catalytic reactions, the applied potentials on materials generally deviate from the equilibrium potentials, being either more positive or more negative. When the applied potentials surpass the redox potentials, it induces instability in the catalytic materials and triggers the oxidation or reduction of surface atoms, consequently altering their valence states. If the restoration of potential is an irreversible process, the reconstructed layer will ultimately comprise genuinely active species [2]. Generally, the structural evolution of catalyst surfaces results in the generation of amorphous or low-crystallinity phases enriched with defects, which significantly boosts their electrocatalytic performance. However, surface reconstruction may also introduce certain adverse effects. For instance, low crystallinity often leads to relatively mediocre conductivity, while an abundance of defects can signify poor structural stability, potentially leading to structural collapse [3].Moderate surface reconstruction has been shown to markedly improve both catalytic activity and stability. Consequently, a comprehensive understanding of the surface reconstruction in catalysts is essential for optimizing their performance and durability.

The phenomenon of surface reconstruction in catalysts is inherently complex and continuously evolving, with the entire reconstruction occurring over an exceedingly brief timescale. Consequently, conventional ex situ characterization techniques are inadequate for accurately identifying the genuine active sites and fail to provide insightful information essential for elucidating the reaction mechanism. To address these limitations, in situ characterization techniques have emerged, enabling the non-destructive monitoring of reaction processes through the detection of reaction intermediates, thereby elucidating the reconstruction behavior and catalytic mechanisms of the catalyst [4]. The advancement of sophisticated in situ characterization techniques, enabling real-time monitoring of catalyst surface states and the evolution of atomic/electronic structures, carries profound implications. These developments are instrumental in elucidating the reaction mechanisms of catalysts, thereby facilitating the design of high-performance catalytic materials [5]. Building upon this foundation, further regulation of the surface reconstruction process can be achieved, leading to the synergistic enhancement of both catalytic activity and stability.

In recent years, in situ testing methods for investigating the surface reconstruction of water-splitting hydrogen evolution catalysts have garnered significant research attention, with new characterization techniques continuously emerging. However, there remains a notable gap in comprehensive review literature on this topic. Liu et al. [6] reviewed recent advances in transition metal-based electrocatalysts for the OER, with a particular focus on identifying the true active species. However, their analysis primarily relied on ex situ characterization techniques, which only capture structural changes before and after the reaction and fail to provide dynamic information on surface reconstruction. Chen et al. [4] summarized recent progress on catalyst reconstruction during OER, highlighting the relationship between structure, surface reconstruction, and intrinsic activity. Nevertheless, similar to most review articles, their discussion was limited to the OER, whereas catalysts involved in the HER may also undergo surface reconstruction. Capturing the dynamic evolution of catalysts during HER using in situ techniques is of great significance for the rational design of high-performance HER electrocatalysts. Moreover, current literature still lacks a comprehensive overview of in situ characterization techniques specifically tailored to monitor surface reconstruction. Therefore, this review seeks to systematically analyze the underlying causes of surface reconstruction, categorize its various types, and explore advanced in situ characterization techniques to provide a detailed understanding of the reconstruction process in water-splitting catalysts. By synthesizing these insights, this review aims to offer valuable perspectives for researchers interested in the surface reconstruction of electrocatalysts.

## 2. Surface Reconstruction

The magnitude of overpotential, reaction conditions, and the intrinsic structural properties of pre-catalysts are crucial factors in modulating the redox transitions of electrocatalysts, thereby significantly impacting their overall catalytic performance [7]. The surface reconstruction of pre-catalysts is predominantly governed by two principal factors: the applied voltage bias during catalytic reactions, as well as the specific testing conditions employed [5]. The first factor encompasses the surface oxidation/reduction phenomena observed in pre-catalysts, which are directly attributable to the application of oxidizing or reducing potentials [7]. For the OER, most non-precious metal-based materials undergo an irreversible surface reconstruction process, which significantly alters their catalytic properties. Throughout the reconstruction phase, the valence state of the metal ions generally undergoes an elevation [4]. Fan et al. employed in situ and ex situ characterization methods to monitor the reconstruction of amorphous CoS_x_ during the OER. Their study revealed the gradual transformation of the Co(OH)_2_ intermediate (CoS_x_ → Co(OH)_2_ → CoOOH), ultimately leading to the formation of CoOOH and achieving outstanding OER performance [8]. Furthermore, multiple studies have indicated that pre-catalysts can undergo surface reconstruction under HER conditions, resulting in localized atomic reconfiguration and the diminution of high-valence metal cations [9]. The second major factor includes temperature, pressure, pH, and solution concentration, among others. These parameters influence the redox potential of the catalyst, thereby affecting the occurrence of the surface reconstruction process. It has been confirmed that higher solution concentration or temperature can facilitate surface reconstruction [10]. Therefore, the applied voltage bias and experimental conditions play a crucial role in governing the surface reconstruction process. Catalysts undergo different surface reconstruction processes under different conditions, which can be mainly divided into four categories: surface reconstruction under alkaline OER conditions, surface reconstruction under acidic OER conditions, surface reconstruction under alkaline HER conditions, and surface reconstruction under acidic HER conditions.

**Figure 1:**
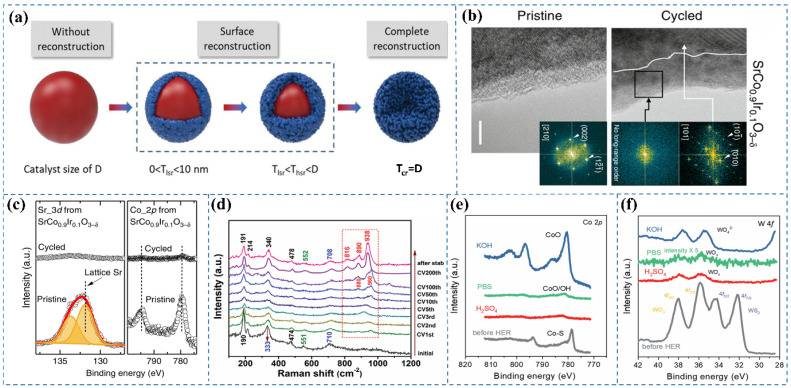
(**a**) Schematic representation of the structural evolution and varying degrees of surface reconstruction in spherical pre-catalysts and their derived reconstructed forms. D indicates the pre-catalyst diameter, while Tlsr, Thsr, and Tcr correspond to the thicknesses of catalysts exhibiting low, high, and full surface reconstruction, respectively [5]. (**b**) HRTEM images depict SrCo_0.9_Ir_0.1_O_3−δ_ before and after undergoing five electrochemical cycles (scale bar: 5 nm) [12]. (**c**) XPS analysis of Sr 3d and Co 2p core levels in SrCo_0.9_Ir_0.1_O_3−δ_ before and after electrochemical evaluation [12]. (**d**) Pseudo-in situ Raman spectra acquired during the HER [13]. (**e**) Co 2p XPS spectra of Co_0.5_W_0.5_S_x_ following HER in 1 M KOH, 1 M PBS, and 0.5 M H_2_SO_4_ electrolytes [14]. (**f**) W 4f XPS spectra of Co_0.5_W_0.5_S_x_ post-HER in 1 M KOH, 1 M PBS, and 0.5 M H_2_SO_4_ electrolytes [14].

Based on the degree of reconstruction in pre-catalysts, the reconstruction results can be classified into three distinct categories: absence of reconstruction, surface-level reconstruction, and full reconstruction. Taking a spherical pre-catalyst with a diameter D as an example (Figure 1a), in the absence of any measurable thickness in the reconstructed layer, no reconstruction occurs. If the entire pre-catalyst transforms into a new species, it is classified as a complete reconstruction process. Conversely, if the thickness of the reconstructed layer (Tsr) is less than D, the process is referred to as surface reconstruction [5]. The extent of reconstruction serves as a critical indicator of the conversion efficiency of pre-catalysts. By optimizing the reconstruction degree, a greater proportion of pre-catalyst components can be transformed into active species, resulting in a more pronounced reconstructed layer, an elevated concentration of active sites, along with improved pre-catalyst utilization efficiency [9]. Muhammad Imran Abdullah et al. [11] reveal that IrO_2_ surface reconstruction markedly increases active site density, intensifies Ir-O bond covalency, and fortifies engagement with oxygen intermediates (*OH, *O, and *OOH), leading to a notable enhancement in the OER efficacy of Ir-based catalysts. However, this reconstruction process may also induce surface amorphization, with the participation of lattice oxygen (LOM) in the OER process, which can lead to excessive oxidation and dissolution of Ir. Macroscopically, this phenomenon results in the degradation of catalytic activity during prolonged operation at high potentials.

### 2.1. OER in Alkaline Conditions

Under alkaline conditions, the surfaces of OER catalysts commonly undergo dynamic transformations, encompassing the formation of hydroxy species, oxidation, and amorphization. These transformations can facilitate the generation of active sites with augmented catalytic activity, thereby exerting a substantial influence on the overall performance of the catalyst. Recently, 3d transition metal nitrides (TMNs) have emerged as efficient catalysts for the OER process. It is noteworthy that the surfaces of TMNs are prone to oxidation, resulting in the formation of oxides and hydroxides. Liu et al. [15] demonstrated that the cobalt oxide/nitrides present on the surface of the synthesized CoVFeN underwent conversion to cobalt (oxy)hydroxide species (CoO_x_(OH)_y_) through surface reconstruction and phase transition during the OER process. Wu et al. [16] revealed that phosphide ions in CoP nanoparticles oxidize to form polyphosphate-like species during the OER, eventually dissolving into the electrolyte, while the catalyst surface transforms into hydroxide/oxide-like species. The restructuring phenomenon is not confined to the surface but may extend deeper into the nanoparticles, potentially leading to the complete alteration of the original CoP structure, particularly in smaller nanoparticles. Moreover, their investigations revealed that CoP nanoparticles can undergo oxidation at potentials below the theoretical threshold for water oxidation. Pathak et al. [17] successfully fabricated a high-performance OER catalyst, Ni_2_P/NiSe_2_@MXene/NF, which exhibited a current density of 10 mA cm^−2^ at a low overpotential of 241.9 mV. To elucidate the morphological evolution and surface reconstruction of the catalyst after electrochemical testing, a series of physicochemical characterizations were conducted. The XRD peak at 21.2° and the broad Raman band observed between 400–585 cm^−1^ were unambiguously assigned to NiOOH species [18,19,20], demonstrating that the catalyst underwent substantial surface reconstruction during the OER process, ultimately forming oxyhydroxide species as the genuine active species.

### 2.2. OER in Acidic Conditions

The reconstruction of catalysts, involving surface and subsurface atomic rearrangement, phase transformation, compositional modification, intermediate adsorption, defect formation, and oxidation of metals or metal compounds, represents a spontaneous alteration in the morphology, geometric structure, and electronic properties of pristine materials (i.e., pre-catalysts) under anodic potential in acidic OER. The reconstruction process in acidic OER catalysts can be classified as either reversible or irreversible. The extent of this reconstruction is largely governed by the physicochemical properties of the pre-catalyst, local pH gradient distribution, applied potential, electrolyte ion type and concentration, and external fields. Notably, the impact of reconstruction on catalytic performance varies across different catalyst types, yielding effects that range from enhancement to deterioration [21]. Wu et al. [16] explored the OER activity of CoP nanoparticles in 0.5 M H_2_SO_4_, noting the swift dissolution of the oxidized surface layer in the acidic environment, which revealed the underlying CoP surface. Trace amounts of phosphate or (hypo)phosphite species were consistently detected on the nanoparticle surface under all experimental conditions. Lin et al. [22] investigated the OER behavior of the Ru/MnO_2_ catalyst under acidic conditions. Their study revealed that RuO_x_ nanoclusters underwent dissolution into the electrolyte, followed by the subsequent redeposition of dissolved Ru ions onto the MnO_2_ matrix, which occurred concurrently with the partial leaching of Mn species. Chen et al. [12] investigated the behavior of the pseudocubic SrCo_0.9_Ir_0.1_O_3−δ_ catalyst, characterized by a corner-shared IrO_6_ octahedral orthorhombic structure, under acidic OER conditions. HRTEM images (Figure 1b) revealed that after five electrochemical cycles, the thickness of surface amorphization on SrCo_0.9_Ir_0.1_O_3−δ_ increased from an initial range of 1–3 nm to approximately 10 nm, indicating significant surface reconstruction during cycling in acidic electrolytes. Furthermore, XPS analysis (Figure 1c) demonstrated that both Sr and Co cations in SrCo_0.9_Ir_0.1_O_3−δ_ underwent substantial leaching following the electrochemical tests.

### 2.3. HER in Alkaline Conditions

It has been observed that catalyst metal species can undergo reduction to lower-valence states when subjected to negative potential environments [9]. In the HER, oxides or hydroxides may also serve as active phases; however, most of these species remain stable only at low overpotential [23]. Wang et al. [13] investigated the surface structural transformation of c-Ni_2_P_4_O_12_/a-NiMoO_x_ during the HER in the alkaline conditions; pseudo-in situ Raman spectroscopy was employed. After the first CV cycle, characteristic peaks appeared at 880 cm^−1^ and 960 cm^−1^, attributed to Mo–O/P–O and PO_4_^3−^ vibrations, respectively (Figure 1d). These spectral features suggest the removal of the surface passivation layer and the initiation of an activation process. With increasing CV cycles, the peak intensities gradually enhanced, while a Mo–O–Ni stretching mode emerged at 910 cm^−1^. Notably, the Raman peak positions remained constant beyond 200 CV cycles, indicating the stabilization of the surface composition. After prolonged stability testing, significant changes in the composition of c-Ni_2_P_4_O_12_/a-NiMoO_x_ were observed, with new peaks at 816, 890, and 938 cm^−1^ attributed to Mo–O vibrations in a-NiMoO_4_. This implies that the nanosheets formed on the c-Ni_2_P4O_12_/a-NiMoO_x_ surface predominantly consist of low-crystalline or amorphous a-NiMoO_4_. Furthermore, Raman signals associated with a-NiMoO_x_ species (191, 214, and 340 cm^−1^) exhibited a blue shift compared to their pre-HER counterparts, suggesting an interaction between the newly formed a-NiMoO_4_ and the existing a-NiMoO_x_. Wu et al. [16] investigated the structural evolution of CoP nanoparticles during alkaline HER catalysis. Under ambient exposure, spontaneous surface oxidation of CoP occurs. When immersed in 1 M KOH solution at open-circuit potential, surface polyphosphate anions undergo hydroxide ion exchange, resulting in a hydroxyl-terminated interface. During HER operation, simultaneous electrochemical reduction of oxidized phosphorus and cobalt species occurs, accompanied by polyphosphate dissolution into the electrolyte, ultimately yielding a cobalt-enriched phosphide surface.

### 2.4. HER in Acidic Conditions

Under acidic conditions during the HER, pre-catalysts commonly reduce to their metallic elemental states due to the highly reductive environment and low pH. Gaining insight into this transformation is crucial for developing efficient and stable HER catalysts in acidic media. Fan et al. [14] demonstrated that, analogous to observations in alkaline electrolytes, the Co 2p XPS peaks underwent a shift toward higher binding energies following HER in both neutral PBS and acidic H_2_SO_4_ electrolytes, indicative of Co–S bond transformation into Co–O bonds (Figure 1e). Concurrently, the intensity of Co XPS peaks significantly diminished, suggesting potential dissolution of the formed Co oxides. Distinctively, in the post-HER state, the W 4f XPS spectra exhibited only two peaks at 37.9 and 35.9 eV, characteristic of 4f_5/2_ and 4f_7/2_ from non-stoichiometric WO_x_ (x < 3), respectively (Figure 1f). This trend differed from that in alkaline electrolytes, pointing to surface alteration of WS_2_ and WO_3_ reduction. The team subsequently prepared Co_0.5_W_0.5_S_x_ as a pre-catalyst, demonstrating pH-sensitive bulk and surface structural transformations during HER. In acidic electrolytes, Co sulfides maintained their initial state, while surface Co sulfides were converted to CoO/Co(OH)_2_ across all pH conditions. Simultaneously, bulk W sulfides converted into highly distorted WO_x_ with lower W oxidation states in electrolytes of varying pH.

## 3. In Situ Characterization of Surface Reconstruction

### 3.1. In Situ Raman

**Figure 2:**
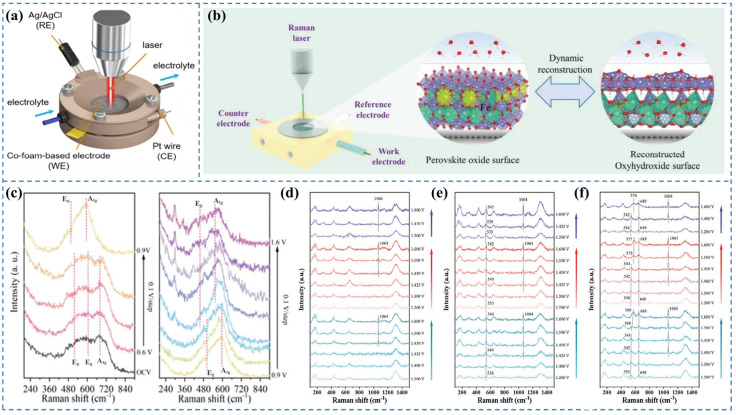
(**a**) Schematic illustration of the in situ electrochemical Raman spectroscopy setup [3]. (**b**) Schematic of the custom-designed operando electrochemical cell for Raman tracking [28]. (**c**) In situ Raman spectra of Cu_2_S/CoFe LDH recorded at 0.1 V intervals [27]. In situ Raman spectra of (**d**) LaFeO_3_, (**e**) La_0.95_Ce_0.05_FeO_3_, and (**f**) La_0.9_Ce_0.1_FeO_3_ under applied potentials [28].

In situ Raman spectroscopy is capable of providing critical information regarding chemical bond transformations of catalysts under reaction conditions and the dynamic reconstruction of active species [5]. It enables non-destructive detection of molecular microstructure information at electrode surfaces/interfaces during liquid-phase electrochemical processes [24]. Owing to its excellent molecular specificity [24], Raman spectroscopy exhibits high selectivity toward low-frequency vibrational modes, such as M–OH, M–OH_2_, and M=O. By monitoring changes in these vibrational features in real time via in situ Raman measurements, the evolution of surface reconstruction can be effectively tracked. In situ Raman spectroscopy is also applicable for identifying reaction intermediates formed during the OER [4]. Moreover, Raman spectroscopy is compatible with diverse environments and can be flexibly integrated with electrochemical cells for in situ measurements [25]. However, its limitations include low spatial resolution and an uncertain probing depth [26]. The schematic representation of this method is illustrated in (Figure 2a).

Wang et al. [27] synthesized a high-performance OER catalyst, Cu_2_S/CoFe LDH, and explored the real-time transformation of the active species during the OER process using in situ Raman spectroscopy. Their analysis indicated that with the elevation of the applied potential, the OER reaction progressed, causing the active components to convert into CoO_2_. The in situ Raman spectra (Figure 2c) demonstrated that upon immersing the catalyst in KOH, three distinct Raman peaks were observed at open-circuit potential, corresponding to the A1g main band of Co_3_O_4_ and the Eg vibrational modes of CoOOH and Co(OH)_2_. Within the lower potential range 0.6–0.8 V (vs. RHE), the Raman peaks remained unchanged, indicating that no transformation of the active phases occurred in this potential regime. At an applied potential of 0.9 V (vs. RHE), the Raman peak corresponding to Co_3_O_4_ vanished, and distinct Raman signals appeared at 508 and 590 cm^−1^, identified as the Eg and A1g vibrational modes of Co–O in CoOOH, respectively, indicating the full transformation of Co_3_O_4_ to CoOOH. When the potential was elevated to 1.2 V (vs. RHE), two distinct Raman bands were observed at 470 and 570 cm^−1^, attributed to the Eg and A1g vibrational modes of CoO_2_, respectively. This indicates that the catalyst Cu_2_S/CoFe LDH underwent transformation into CoO_2_ as the active species during the OER under progressively higher potentials.

To investigate the dynamic structural evolution of the catalyst and identify the actual active species during the OER, Zhai et al. [28] employed a customized liquid electrochemical cell coupled with in situ Raman spectroscopy, as illustrated in Figure 2b. In situ Raman spectroscopy of La_1−x_Ce_x_FeO_3_ (Figure 2d–f) revealed the emergence of the *α*-FeOOH peak at 1064 cm^−1^ when the anodic potential was increased to 1.45 (vs. RHE) [29]. Notably, the α-FeOOH peak completely disappeared upon returning the potential to 1.2 (vs. RHE) and reappeared when the potential was reapplied at 1.45 (vs. RHE), indicating the reversible transformation of La_1−x_Ce_x_FeO_3_ to *α*-FeOOH during the OER process.

### 3.2. In Situ XRD

**Figure 3:**
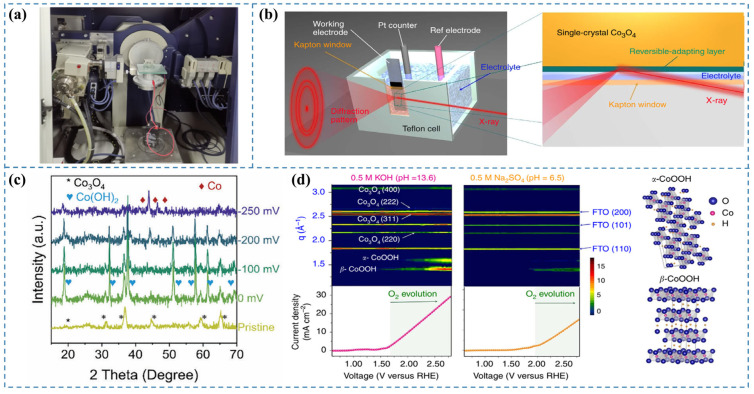
(**a**) Photograph of an XRD diffractometer integrated with a two-electrode electrolysis system [34]. (**b**) Diagram of the in situ GIXRD system integrated with a liquid electrochemical cell [35]. (**c**) In situ XRD patterns for S-Co_3_O_4_/CC recorded during HER [33]. (**d**) Contour maps of in situ GIXRD patterns for a Co_3_O_4_@CoO single crystal in 0.5 M KOH and 0.5 M Na_2_SO_4_ aqueous solutions [35].

X-ray diffraction (XRD) serves as a versatile characterization tool for determining critical structural parameters of materials, including crystalline phase identification and lattice constants. The development of in situ XRD techniques has enabled real-time monitoring of catalyst structural dynamics during electrocatalytic reactions, making it invaluable for mechanistic studies [30,31]. However, this approach faces inherent limitations when analyzing reconstructed catalyst surfaces with low crystallinity or when detecting weak scattering signals at solid-liquid interfaces [32]. A photo of the XRD diffractometer equipped with a two-electrode electrolysis system for in situ XRD measurements is shown in Figure 3a.

To elucidate the surface reconstruction behavior of S-Co_3_O_4_/CC during the alkaline HER process and identify the genuine active species, Fan et al. [33] performed in situ characterization of the catalyst using a liquid electrochemical cell. In situ XRD analysis (Figure 3c) reveals that crystalline Co(OH)_2_ forms under open-circuit potential conditions. Upon applying a reductive potential of −200 mV (vs. RHE), characteristic diffraction peaks of metallic Co (hcp) emerge. The S-Co_3_O_4_/CC catalyst undergoes complete reduction to the Co (hcp) phase at a cathodic potential of −250 mV (vs. RHE). These observations demonstrate the potential-driven reconstruction of S-Co_3_O_4_/CC into metallic Co (hcp) as the genuine active species for the HER.

### 3.3. In Situ Synchrotron GIXRD

Synchrotron radiation sources, known for their exceptionally high intensity and flux, enable the determination of atomic structures at catalyst surfaces under environmental conditions through in situ grazing-angle X-ray diffraction in liquid environments. This capability is instrumental in elucidating surface reconstruction phenomena of water electrolysis catalysts during reaction processes. However, it still faces several limitations, including its applicability primarily to well-ordered crystalline materials, the relatively weak scattering signals from buried solid–liquid interfaces under conventional X-ray sources, and current accessibility constraints associated with synchrotron radiation facilities [32].

Tung et al. [35] aimed to monitor the evolution of surface states of the prepared catalysts during the OER. They designed a custom reaction cell and, leveraging the unique high intensity and flux of synchrotron radiation, implemented in situ GIXRD in aqueous conditions to determine the surface atomic arrangement of the catalyst under OER conditions (Figure 3b). By plotting the LSV curve with applied voltage as the abscissa and color-coding the diffraction intensity recorded using 12 keV synchrotron light at the same voltage coordinates (Figure 3d), they observed the emergence of the β-CoOOH phase beyond a certain voltage threshold, indicating the transformation of the CoO phase into the active species β-CoOOH with increasing voltage. Furthermore, upon further increasing the applied voltage, the α-CoOOH phase was also detected. It is noteworthy that the evolution of oxygen commences concomitantly with the formation of oxyhydroxides. Their study elucidates that under the alkaline conditions of the OER, the catalyst undergoes surface reconstruction, leading to the generation of oxyhydroxides as the genuine active species for the OER process.

### 3.4. In Situ FTIR

**Figure 4:**
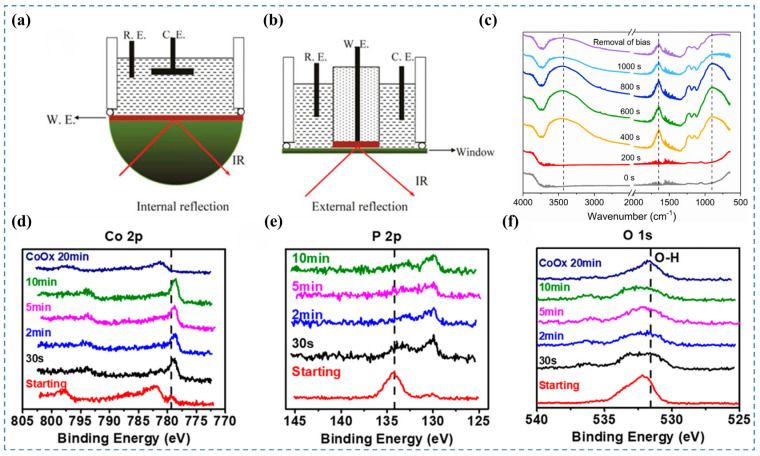
(**a,b**) Simplified schematic of the in situ FTIR experimental setup [4]. (**c**) In situ FTIR spectra of CoS_x_ recorded under a 1 mA anodic current in 1 M KOH [8]. (**d**–**f**) XPS spectra of Co 2p, P 2p, and O 1s core levels [16].

In situ Fourier Transform Infrared (FTIR) spectroscopy is used to identify molecular species present on the catalyst surface during reactions, allowing for the monitoring of active species changes and elucidation of reaction pathways [36]. This technique provides multiple benefits, such as extensive applicability, low sample volume, ease of use, quick analytical processes, and exceptional sensitivity [37]. The experimental in situ FTIR setup is schematically illustrated in Figure 4a and Figure 4b. By irradiating the sample with infrared light of varying frequencies, molecular vibrations and rotations induce changes in dipole moments, resulting in transitions from the ground to excited energy states. The molecular absorption spectrum is obtained by detecting reductions in transmitted light intensity in the absorption region [38]. From this spectrum, characteristic absorption frequencies and peak intensities can be analyzed to qualitatively identify molecular species. As each molecule has a unique infrared spectrum, specific species can be directly recognized [39]. During catalyst surface reconstruction, the precatalyst evolves through intermediate states into the final product. These transformations involve changes in molecular vibrations and rotations, leading to shifts in IR absorption frequencies and corresponding spectral changes. The high sensitivity of in situ FTIR enables the detection of these molecular changes, providing clear evidence of surface reconstruction [4]. However, in situ FTIR spectroscopy still faces several limitations. To implement in situ FTIR effectively in electrochemical systems, multiple challenges associated with enhancing the signal-to-noise ratio (S/N) at the liquid–solid interface must be addressed. These include the strong absorption of infrared light by aqueous electrolytes, energy loss due to infrared reflection at the electrode surface, and the inherently weak infrared signals of sub-monolayer adsorbates [26].

Fan et al. [8] synthesized CoS_x_ catalysts for the OER under alkaline conditions. To investigate the phase transformation of the catalyst during the OER process, they conducted in situ FTIR spectroscopy at a constant current of 1 mA over various reaction durations (Figure 4c). After 200 s, the bands at 3350 and 1630 cm^−1^ showed an upward trend in intensity correlated with extended reaction time. These bands eventually stabilized as the reaction proceeded further. The observed bands are attributed to the stretching and bending vibrations of hydroxyl groups adsorbed on the catalyst surface, indicating an enhanced adsorption of H_2_O during the OER process. At 892 cm^−1^, the enhanced spectral feature corresponds to bending vibrations of hydroxyl groups within the catalyst structure, which demonstrates hydroxide phase evolution on the surface under OER conditions. Furthermore, after conducting the OER for 1000 s, the applied bias was removed to halt the reaction, followed by another FTIR measurement. The persistent bands at 3350, 1630, and 892 cm^−1^ demonstrate that OER-induced structural/compositional modifications in the catalyst are non-reversible.

### 3.5. In Situ XPS

X-ray Photoelectron Spectroscopy (XPS) provides quantitative surface-specific analysis, enabling precise determination of elemental distribution, oxidation states, and local chemical bonding in materials. In situ XPS enables the monitoring of dynamic changes in the chemical composition and oxidation states of catalysts during reactions, thereby providing insights into surface reconstruction processes [4]. However, the requirement for ultrahigh vacuum (UHV) conditions in conventional XPS has posed challenges for its in situ or operando application in monitoring electrochemical processes [26]. Synchrotron-based ambient-pressure XPS, equipped with differential pumping stages, effectively addresses this limitation and enables surface analysis of electrocatalysts under near-operando conditions [40,41].

Wu et al. [16] investigated the dynamic evolution of the CoP catalyst under alkaline HER conditions using in situ XPS. They conducted the HER at a constant potential of −150 mV (vs. RHE) and monitored the changes in the in situ XPS spectra at various reaction times. In the Co 2p spectra (Figure 4d), the Co–O peak at 782.1 eV disappeared, while the intensity of the Co–P peak at 779.3 eV increased and slightly shifted towards lower binding energy. In the P 2p spectra (Figure 4e), the P–Co component at 130.0 eV increased, whereas the P–O component at 134.2 eV decreased. These spectral changes indicate that the majority of the oxide species on the surface of the CoP catalyst can be reduced under alkaline HER conditions. This observation is further corroborated by the significant reduction in the intensity of the O 1s spectra (Figure 4f). Therefore, for alkaline HER catalysts, it is possible that the catalyst surface becomes oxidized at open-circuit potential due to the highly alkaline environment. Upon application of a cathodic potential, the surface oxides may subsequently be reduced.

### 3.6. In Situ XAS

**Figure 5:**
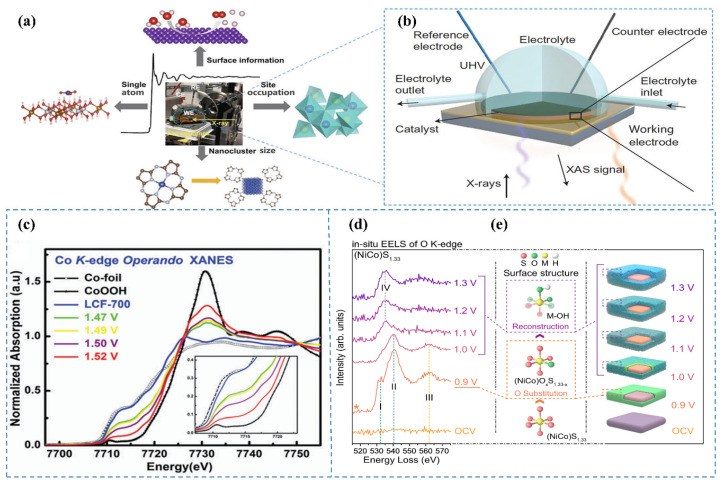
(**a**) The applicable range of in situ XAS [4]. (**b**) Schematic of the in situ XAS configuration for electrocatalyst analysis [4]. (**c**) In situ XAS spectra at the Co K-edge XANES for LCF-700, measured within the potential range of 1.47 to 1.52 V (vs. RHE) in 0.1 M KOH [44]. (**d**) In situ EELS spectra of the O K-edge at varying applied potentials [45]. (**e**) Schematic representation of the sulfur-to-oxygen substitution within the crystal lattice [45].

Owing to its exceptional sensitivity to local electronic configurations and geometric arrangements, X-ray absorption spectroscopy (XAS) has emerged as a formidable technique for precisely monitoring the dynamic structural transformations at the atomic scale under operational conditions [7]. Although in situ XAS enables monitoring of valence state changes during the OER process, its bulk-sensitive nature limits its ability to directly probe specific oxidation states of surface metal sites, providing only averaged information from bulk and surface phases [24,42]. In situ XAS has been widely employed to track variations in the oxidation state and local structure of metal centers in various catalysts, as well as to analyze the formation of transient intermediates from time-resolved spectra, which can help determine whether surface reconstruction occurs [43]. The applicable range of in situ XAS and the experimental setup for in situ XAS characterization of electrocatalysts are illustrated in Figure 5a and Figure 5b, respectively.

To elucidate the genuine active sites of the LCF-700 catalyst during the OER, Song et al. [44] applied in situ XAS to capture the dynamic alterations of the catalyst under reaction conditions. By modulating the applied voltage during OER, they investigated the XANES spectra of the catalyst (Figure 5c). The spectral analysis revealed a shift of the absorption edge toward higher energy as the voltage increased, indicative of an elevation in the oxidation state of cobalt. Notably, at a potential of 1.52 V (vs. RHE), the position of the absorption edge closely resembled that of CoOOH, suggesting the presence of cobalt in a trivalent state. Additionally, they observed analogous transformations in the intensity of the iron metal peak with increasing voltage, implying an augmentation in the oxidation state of iron as well. In situ XAS observations demonstrate that under alkaline OER conditions, the oxidation states of the metals increase with escalating voltage.

### 3.7. In Situ EELS

Electron Energy Loss Spectroscopy (EELS) is an analytical technique based on TEM that measures the energy loss of electrons after their interaction with a sample to obtain information on the atomic structure, chemical composition, and electronic properties of materials. EELS is characterized by its high spatial resolution and sensitivity, making it widely applicable in fields such as materials science, nanotechnology, chemistry, and physics. The fundamental principle of EELS lies in the inelastic scattering of high-energy electrons as they pass through the sample, resulting in energy losses. These energy losses are associated with specific excitation processes within the sample, such as inner-shell electron excitations and plasmon excitations. By analyzing the energy loss spectrum of the electrons, the elemental composition, chemical states, and electronic structure of the sample can be inferred. Sample preparation for EELS in TEM is critical for obtaining reliable and interpretable data. One of the primary objectives during TEM sample preparation for EELS is to ensure that the sample faithfully represents the intrinsic properties of the material without introducing artifacts. For in situ TEM investigations involving reactive materials as electrodes, meticulous care must be taken during sample preparation to minimize the influence of ambient air and moisture. A widely adopted approach involves the use of a glovebox or a customized argon-flow glovebag when mounting the reactive material onto the sample holder and transferring the TEM holder into the microscope. These precautions significantly mitigate side reactions that could otherwise alter the intrinsic characteristics of the sample [46]. Despite its powerful capability for atomic-scale imaging, in situ TEM faces several technical challenges when applied to dynamic processes in liquid environments. First, achieving ultrahigh spatial resolution is hindered by the thickness of the liquid layer, which can scatter electrons and reduce image clarity; this limitation can be mitigated by minimizing the thickness of the liquid film. Second, the relatively low temporal resolution and slow image acquisition rates limit the ability to capture transient intermediate states; the integration of direct electron detection cameras offers a solution by enabling faster frame rates and improved sensitivity. Third, conventional energy-dispersive X-ray spectroscopy (EDS) and EELS suffer from long acquisition times and low signal-to-noise ratios due to limited energy resolution; employing dual-energy-filtered STEM imaging with energy resolutions down to 0.01 eV significantly enhances spectral efficiency and reduces acquisition time [47].

Hu et al. [45] systematically investigated the potential-dependent electronic states of oxygen in (NiCo)S_1.33_ during alkaline OER through in situ EELS characterization. The in situ EELS of the O K-edge (Figure 5d) revealed three distinct peaks (I, II, and III) at 0.9 V, which were assigned to the hybridization of O 2p with Co 3d [48,49], Ni 3d [50,51], and Co 4sp [52,53] orbitals, respectively. These results indicate that oxygen atoms incorporate the surface lattice of (NiCo)S_1.33_ (Figure 5e) by replacing a portion of sulfur atoms and forming ionic bonds with Co and Ni atoms, while the overall sulfide matrix crystal structure of the catalyst was preserved. As the surface reconstruction progressed, the dissociation of the surface crystal structure induced the breaking of Co–O and Ni–O bonds, which was reflected in the evolution of characteristic peaks. Based on this analysis, they concluded that at a potential of 0.9 V, the lattice sulfur atoms on the surface of (NiCo)S_1.33_ were exchanged with oxygen atoms, leading to the formation of an oxygen-sulfur coexisting surface, i.e., (NiCo)O_x_S_1.33-x_. With further increase in voltage, (NiCo)O_x_S_1.33-x_ underwent reconstruction into metal oxy/hydroxides.

### 3.8. In Situ UV-Vis

**Figure 6:**
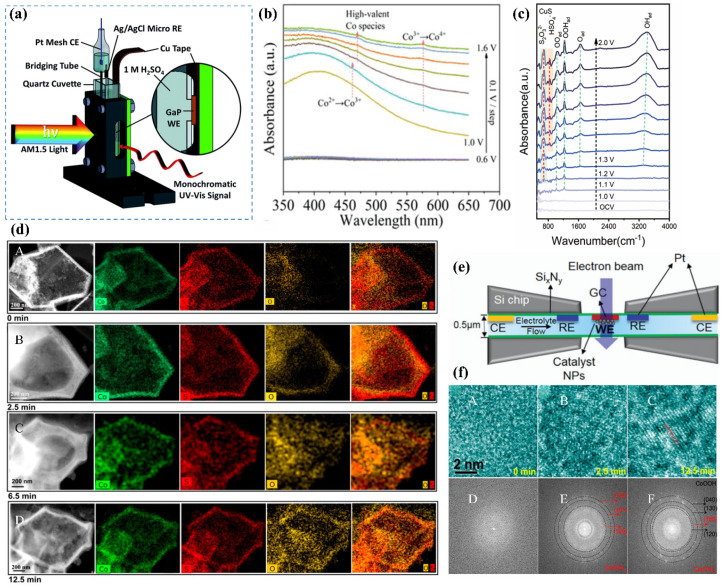
(**a**) Schematic of the in situ UV-vis setup with a three-electrode system [56]. (**b**) In situ UV-vis absorption spectra of Cu_2_S/CoFe LDH, recorded from open-circuit voltage (OCV) to 1.6 V (vs. RHE) in 0.1 V increments [27]. (**c**) ATR-IR spectral analysis of CuS [58]. (**d**) In situ TEM monitoring of CoS_x_ structural evolution during OER; (A–D) HAADF images illustrating the morphological changes in CoSx over time during OER [8]. (**e**) Schematic representation of the in situ TEM setup [59]. (**f**) (A–F) In situ HRTEM analysis revealing the transformation of CoS_x_ into alpha-phase CoOOH during OER [8].

In situ UV-vis (ultraviolet-visible spectroscopy) provides a powerful approach for monitoring the structural evolution of catalysts during electrochemical processes by analyzing the interaction between UV-vis light and surface species on the electrode [5]. The advantages of this technique lie in its non-destructive nature and the ability to reuse samples. In addition, it allows for rapid measurements. However, its accuracy may be limited by measurement errors arising from light scattering. This technique, grounded in electronic transitions, provides a direct means to observe the redox behavior of metal ions within catalytic systems [24]. The underlying principle involves detecting variations in light absorption, which arise from energy differences required for electronic transitions in different molecules, predominantly within the UV and visible spectral ranges [54,55]. A schematic diagram of the in situ UV-vis setup based on a three-electrode system (Figure 6a) [56]. Experimentally, incident light is dispersed into distinct wavelengths, passed through the sample, and the absorption intensity at each wavelength is recorded to generate the UV-vis absorption spectrum [4]. This approach is particularly valuable for investigating changes in the oxidation states of metal species induced by surface reconstruction during catalytic reactions.

Wang et al. [27] fabricated a Cu_2_S/CoFe LDH catalyst through a straightforward electrodeposition technique, wherein CoFe LDH was deposited onto Cu_2_S. The transformation of metal ion oxidation states during pre-catalyst surface reconstruction was investigated using in situ UV-vis spectroscopy. The absorption spectra (Figure 6b) revealed that the UV-vis profiles remained invariant within the potential window of 0.6–0.9 V (vs. RHE), suggesting that the oxidation state of Co remained unaltered in this regime. At an applied potential of 1 V (vs. RHE), a broad absorption feature centered at 460 nm was observed, corresponding to the Co^2+^/Co^3+^ oxidation process [57]. Moreover, when the potential exceeded 1.2 V (vs. RHE), an additional absorption band was observed at 565 nm, which is attributed to the oxidation of Co^3+^ to Co^4+^ during the surface reconstruction of the Cu_2_S/CoFe LDH catalyst [57]. These in situ UV-vis spectroscopic findings unequivocally demonstrate that the pre-catalyst undergoes surface reconstruction during the OER, concomitant with a transition to higher oxidation states of the metal ions.

### 3.9. In Situ ATR-IR

Attenuated total reflectance infrared spectroscopy (ATR-IR) enables real-time monitoring of chemical changes on the catalyst surface during reactions. For instance, in electrocatalytic processes, ATR-IR can detect infrared absorption features corresponding to adsorbed reactants, transient intermediates, and final products, providing insights into reaction progress and surface species evolution. This technique offers high sensitivity and is non-destructive to samples. However, its application is limited by relatively low spectral resolution.

Zhu et al. [58] performed in situ ATR-IR spectroscopy on the prepared CuS catalyst to investigate its dynamic reconstruction behavior during the OER process. A continuous voltage ranging from 0 to 2 V was applied to drive the OER reaction while simultaneously collecting infrared spectra. As shown in the infrared spectrum of CuS (Figure 6c), characteristic peaks of S_2_O_3_^2−^ and HSO^4−^ located at 673 cm^−1^ and 850 cm^−1^ were observed, indicating the oxidation of S^2−^ to sulfur oxide anions during the OER process [60]. Additionally, the emergence of the OOHad peak suggests the presence of metastable Cu III oxide species during the reconstruction process, which are considered to be catalytically active substances for the OER.

### 3.10. In Situ TEM

In situ transmission electron microscopy (TEM) offers a powerful approach to directly visualize catalytic processes and the dynamic evolution of catalysts. Catalysis, inherently a dynamic phenomenon, encompasses key steps such as molecular adsorption, activation, and desorption. To capture the surface reconstruction of catalysts and the evolution of active sites, real-time observation with precise temporal resolution is essential. TEM provides structural and chemical information on electrode materials at the nanoscale. In situ TEM facilitates real-time observation of the morphological and structural evolution of electrocatalysts under operating conditions [26]. However, several limitations exist for in situ TEM, including reduced spatial resolution due to electron scattering from the liquid electrolyte in windowed cells, beam-induced alterations to the catalyst structure and performance, and the inherent difficulty in detecting transient reaction intermediates [61]. Additionally, the requirement for high-vacuum environments due to the high-energy electron source imposes strict constraints on the design and operational conditions of miniaturized in situ cells [62]. In situ TEM sample preparation typically involves isolating post-electrochemically tested materials from a glassy carbon rotating disk electrode (utilized in conventional three-electrode systems) onto carbon-supported TEM grids. During CV, morphological changes were monitored using in situ TEM [63]. This conceptual framework depicts the in situ TEM experimental setup (Figure 6e) [59]. 

Fan et al. [8] utilized in situ TEM to investigate the dynamic evolution of CoS_x_ catalysts during the alkaline OER. The study monitored morphological changes under a maintained current density of 10 mA over varying reaction durations. Initially, the CoS_x_ material exhibited a well-defined hollow morphology with an amorphous structure (Figure 6(dA)). After 2.5 min of OER (Figure 6(dB)), the thickness of the surface shell increased significantly from 45 nm to 115 nm. The confined cavity within the hollow structure limited oxygen release, leading to the accumulation of OOH* intermediates, which progressively etched the inner shell of CoS_x_, forming Co(OH)_2_ or CoOOH nanocrystals. The procedure induced the development of a dense oxygen-enriched internal layer, with structural evolution captured in (Figure 6(dC)). Eventually, the outer shell succumbed to the high internal pressure of encapsulated oxygen, causing it to rupture (Figure 6(dD)) or even collapse [64]. Analysis of in situ high-resolution TEM (HRTEM) images revealed the amorphous nature of the initial material (Figure 6(fA,D)). However, after 2.5 min of anodic oxidation, highly dispersed nanocrystals nucleated within the amorphous CoS_x_ shell (Figure 6(fB,E)). Extending the electrochemical treatment to 12.5 min revealed well-defined CoOOH crystallographic features (Figure 6(fC,F)), demonstrating phase evolution from amorphous CoS_x_ through Co(OH)_2_ intermediates to crystalline CoOOH.

### 3.11. EC-AFM

**Figure 7:**
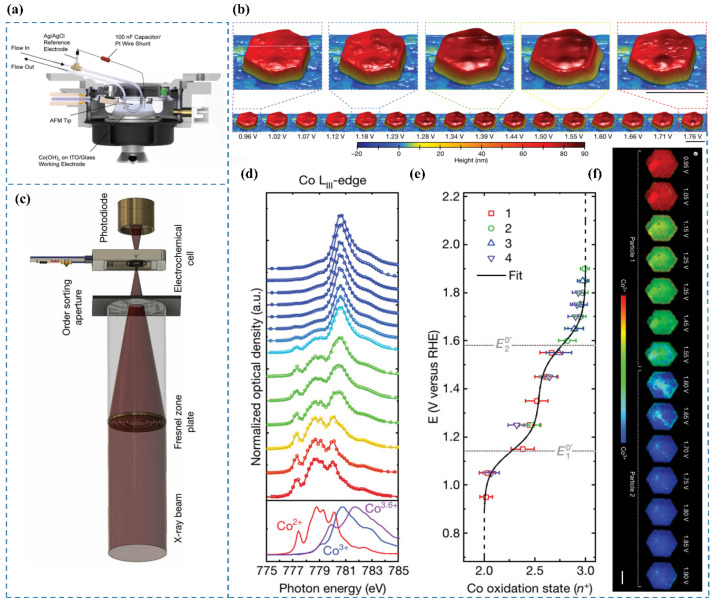
(**a**) Schematic of the operando EC-AFM cell [65]. (**b**) Surface morphology of a β-Co(OH)_2_ particle in 0.1 M KOH under varying applied potentials. Scale bars: 500 nm [65]. (**c**) Experimental configuration of the electrochemical flow cell integrated with the STXM microscope [65]. (**d**) Co LIII-edge STXM-XAS spectra averaged across particles as a function of applied potential [65]. (**e**) Correlation between applied potential and the oxidation state of Co [65]. (**f**) Phase maps illustrating the steady-state oxidation states of Co in β-Co(OH)_2_ particles 1 and 2 at different potentials. Scale bar: 1 μm [65].

Atomic force microscopy (AFM) is also a widely used surface characterization technique that enables nanoscale-resolution imaging of surface morphology, structure, and roughness. Surface reconstruction of precatalysts is often accompanied by changes in topography and structure, which can be directly and intuitively captured in real time using AFM [25]. Its major limitation lies in the complexity of operation. Electrochemical atomic force microscopy (EC-AFM) enables real-time imaging and precise monitoring of nanoscale surface morphology alterations within controlled electrochemical environments [65]. This is the Operando EC-AFM Cell (Figure 7a).

Chueh et al. [65] employed EC-AFM [66,67,68,69] to investigate voltage-induced morphological transformations of particles in 0.1 M KOH at a spatial resolution of approximately 10 nm. The experimental cell configuration is illustrated in Figure 7a. Initial observations at E = 1.12 V revealed heterogeneous nucleation of expanded regions within the particle, which propagated radially toward both the interior and the edges. Within the voltage range of 1.3 V to 1.5 V, the particle exhibited an expanded hexagonal core encircled by a hexagonal ring approximately 100 nm thick, maintaining a constant height. Further voltage elevation triggered particle contraction, initiating from the outer edges and progressing inward toward the center (Figure 7b).

### 3.12. STXM

Scanning transmission X-ray microscopy (STXM) is an advanced spectroscopic imaging technique that integrates third-generation synchrotron radiation facilities, high-intensity laboratory X-ray generators, and precision X-ray focusing systems. By utilizing transmission X-ray absorption contrast mechanisms, this method achieves three-dimensional visualization with nanoscale spatial resolution while simultaneously generating chemical composition data. Although STXM offers important insights into the spatially resolved reconstruction behavior of catalysts, its application under in situ conditions is often limited by insufficient spatial resolution and sensitivity, which pose significant challenges for directly visualizing atomic rearrangements during the formation of reconstructed species [70,71].

**Figure 8:**
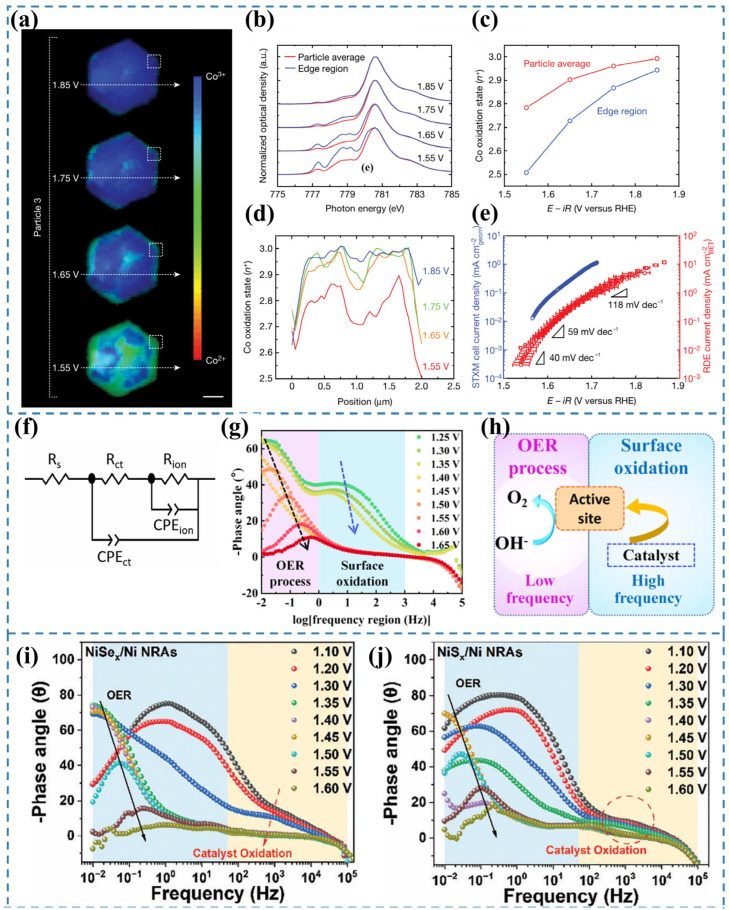
(**a**) Phase distribution maps of a β-Co(OH)_2_ particle under varying applied potentials across distinct OER Tafel regions [65]. (**b**) Co LIII-edge STXM–XAS spectra [65]. (**c**) Derived Co oxidation states for the entire particle and edge region, extracted from the Co LIII-edge XAS spectra in (**b**) [65]. (**d**) Line profiles of Co oxidation states along the trajectories indicated in (**a**) [65]. (**e**) Comparative OER Tafel plots obtained from the STXM cell and a conventional RDE cell at a scan rate of 10 mV s^−1^ [65]. (**f**) Equivalent circuit employed to simulate the measured electrochemical response [75]. (**g**) In situ Bode phase plots of NiMOF [76]. (**h**) Illustration of the OER process taking place at the electrode–electrolyte boundary with respect to EIS analysis [76]. (**i**) Bode plots of in situ EIS of NiSe_x_/Ni NRAs [77]. (**j**) Bode plots of in situ EIS of NiS_x_/Ni NRAs [77].

Chueh et al. [65] conducted operando STXM measurements at the Co LIII-edge to determine the local oxidation state of cobalt within particles, achieving a spatial resolution of 50 nm [72]. The experimental setup is shown in Figure 7c. By averaging single-pixel STXM–XAS measurements across individual particles, they derived the particle-averaged XAS spectrum (Figure 7d). The relationship between the Co oxidation state and applied voltage is illustrated in Figure 7e. Additionally, nanoscale phase maps (Figure 7f) were generated to visualize the local Co oxidation state with 50 nm resolution. Their findings revealed a direct correlation between the Tafel slope and the local Co oxidation state (Co^3+^ coverage) at reactive edge sites (Figure 8). Based on operando STXM data and microkinetic modeling [73], at 1.55 V, a low coverage of CoOOH species with an average cobalt oxidation state of approximately +2.5 was observed at the edges, which was associated with high catalytic activity, as indicated by a Tafel slope of 40 mV dec^−1^. With increasing potential, the CoOOH coverage gradually reached saturation (oxidation state close to +3) at potentials above 1.75 V, leading to a significantly higher Tafel slope of 120 mV dec^−1^. The results indicate that Co^3+^ in β-CoOOH acts as the active site for the OER [74].

### 3.13. In Situ EIS

In situ electrochemical impedance spectroscopy (EIS) is a powerful tool for investigating the kinetic behavior of electrochemical reactions and probing the dynamic evolution of reactive species at active sites [78]. In situ EIS enables real-time monitoring of catalytic reaction kinetics and electrode–electrolyte interfacial properties. It also allows the observation of impedance changes during the reconstruction process and provides insights into the correlation between microstructural evolution and electrochemical performance [79]. Owing to its operational simplicity and effectiveness, this technique holds great potential to attract increased attention in the study of catalyst reconstruction [5]. To accurately quantify the charge transfer and ion diffusion resistances of the reaction system, the equivalent circuit model shown in Figure 8f was employed for fitting analysis [75]. The equivalent circuit model comprises two distinct components. The first component, denoted as Rs, corresponds to the electrolyte resistance. The second component describes the electrochemical processes occurring at the electrode–electrolyte interface, which include two parallel RC circuit elements. The first RC pair consists of CPEct and Rct, representing the double-layer capacitance and charge-transfer resistance associated with the Faradaic reaction, respectively [80,81]. The second RC pair, involving CPEion and Rion, accounts for the pseudocapacitive behavior and ion diffusion resistance arising from the adsorption and desorption of reaction intermediates at the interface [82,83].

Bode plots are employed to characterize the frequency-dependent phase angle behavior. A larger phase angle indicates more capacitive-like characteristics in the equivalent circuit, suggesting greater electron participation in double-layer capacitance formation while fewer charges are involved in Faradaic processes [75]. The peak observed in the high-frequency region is attributed to the pre-oxidation of the electrocatalyst, while the low-frequency peak originates from the onset of the OER (Figure 8g,h) [76]. The magnitude of phase angle variation in the high-frequency region reflects the degree of surface reconstruction of the catalyst. Wang et al. [77] employed in situ EIS and Raman spectroscopy to probe the dynamic evolution of NiA_x_ (A = S, Se) during the OER. For NiSe_x_/Ni NRAs (Figure 8i), a sharp decrease in the high-frequency phase angle was observed within the potential range of 1.10–1.35 V (vs. RHE), indicative of profound oxidative reconstruction of NiSe_x_ accompanied by extensive Se^2−^ oxidation. When the potential exceeded 1.40 V (vs. RHE), the high-frequency phase angle reached a plateau, followed by a rapid decrease in the low-frequency region, marking the onset of OER activity. These observations were further corroborated by in situ Raman spectroscopy, which detected characteristic NiOOH vibrational modes emerging at potentials ≥ 1.40 V (vs. RHE), confirming the oxidative reconstruction of NiSe_x_. In contrast, NiS_x_/Ni NRAs (Figure 8j) exhibited significantly weaker phase angle variations in the high-frequency region compared to their NiSe_x_ counterparts, suggesting a more limited degree of oxidative reconstruction under identical conditions. Therefore, in situ EIS provides critical insights into the catalyst’s surface reconstruction dynamics, but it cannot unambiguously identify the final active species. Complementary characterization techniques are required for further verification.

### 3.14. In Situ DEMS

Isotope labeling has emerged as a powerful approach for elucidating the mechanistic pathways of OER electrocatalysis. This technique is often integrated with analytical methods including mass spectrometry, Raman spectroscopy, and infrared spectroscopy to enable the detection and identification of key reaction intermediates. In particular, ^18^O labeling provides critical insight into O–O bond formation and facilitates the differentiation of mechanistic routes. Unlike the adsorbate evolution mechanism (AEM), the lattice oxygen mechanism (LOM) is distinguished by the direct participation of lattice oxygen in the catalytic cycle, offering a distinct diagnostic advantage in isotope-based mechanistic studies [84]. Differential Electrochemical Mass Spectrometry (DEMS) is an advanced characterization technique that combines electrochemical testing with real-time mass spectrometry (MS) to monitor dynamic gaseous products during electrochemical reactions. This provides unique insights into reaction mechanisms.

**Figure 9:**
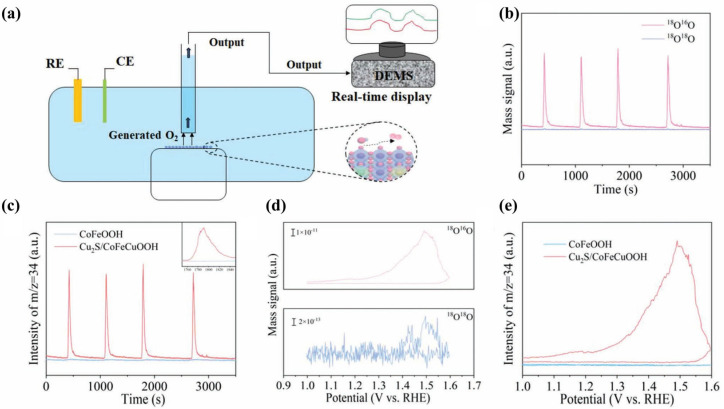
(**a**) Schematic illustration of the in situ ^18^O isotope-labeled DEMS setup for detecting evolved O_2_ [27]. (**b**) Time-dependent DEMS signals of ^34^O_2_ and ^36^O_2_ recorded for Cu_2_S/CoFeCuOOH [27]. (**c**) Comparative time-resolved ^34^O_2_ signals for CoFeOOH and Cu_2_S/CoFeCuOOH [27]. (**d**) Potential-dependent evolution profiles of ^34^O_2_ and ^36^O_2_ signals for Cu_2_S/CoFeCuOOH [27]. (**e**) ^34^O_2_ signal variations as a function of applied potential for both CoFeOOH and Cu_2_S/CoFeCuOOH [27].

Wang et al. [27] employed in situ ^18^O isotope-labeled DEMS to identify the active sites involved in the OER. A schematic illustration of the experimental setup for tracking O_2_ evolution via ^18^O labeling is shown in Figure 9a. In this approach, the electrocatalyst was first labeled with ^18^O, followed by cyclic voltammetry (CV, 1.0–1.6 V vs. RHE) in a ^16^O-containing electrolyte, during which the evolved oxygen species were continuously monitored. For CoFeOOH, no signal corresponding to ^18^O^16^O (*m*/*z* = 34) was detected (Figure 9c,e), indicating that lattice oxygen was not involved in the OER. In contrast, a distinct ^18^O^16^O peak was observed for the Cu_2_S/CoFeCuOOH catalyst (Figure 9b,d), confirming the participation of lattice oxygen. Based on these results, along with complementary characterizations and theoretical calculations, the authors concluded that although both CoFe LDH and Cu_2_S/CoFeCu LDH undergo surface reconstruction, the CoFeCuO_2_ phase derived from Cu_2_S/CoFeCuOOH possesses a unique electronic structure that enables the formation of oxygen-active sites concurrent with surface reconstruction. Zhao et al. [85] demonstrated that surface reconstruction under OER conditions could shift the reaction mechanism from LOM to AEM, as evidenced by DEMS analysis alongside other techniques.

## 4. Summary and Outlook

Surface reconstruction of electrocatalysts represents a highly intricate process, the evolution of which is challenging to elucidate through conventional ex situ characterization methods. In situ characterization techniques offer a powerful approach to capture transient reaction intermediates, identify active sites, and monitor dynamic structural and compositional changes in catalysts. This review comprehensively examines the surface reconstruction behavior of electrocatalysts under diverse conditions, including the OER and HER in both alkaline and acidic environments. Additionally, it provides an in-depth overview of recent progress in in situ characterization techniques utilized to study surface reconstruction phenomena, including in situ Raman, in situ XRD, in situ GIXRD, in situ FTIR, in situ XPS, in situ XAS, in situ UV-vis, in situ EELS, in situ ATRIR, in situ TEM, EC-AFM, STXM, in situ EIS, and in situ DEMS.

Despite significant advancements in in situ characterization methodologies, more systematic investigations are essential to address existing challenges and further advance the field.

(1) The reliance on a single in situ characterization technique is insufficient to comprehensively elucidate the phase, valence state, structure, and composition of catalysts. Consequently, integrating multiple in situ characterization methods is essential to provide a holistic understanding of catalyst evolution and to accurately identify the catalytically active components.
(2) The application of in situ characterization techniques to reveal the surface reconstruction process of electrocatalysts remains a complex endeavor. A significant challenge lies in reducing the technical complexity and operational difficulty associated with in situ testing. Innovations in experimental design, instrumentation, and data analysis are critical to streamline these processes and enhance their accessibility.
(3) Many in situ characterization methods require vacuum conditions, which starkly contrast with the realistic environments where electrocatalyst surface reconstruction occurs. Therefore, advancing techniques that operate under ambient or reaction-relevant conditions represents a crucial development direction. Such advancements will enable a more accurate depiction of surface reconstruction processes under practical scenarios.
(4) Certain characterization methods are limited by their low radiation energies, which restrict their application to materials with strong signal responses. Synchrotron radiation, characterized by its exceptionally high energy, offers a transformative solution by enabling the detection of robust signals even in low-doping materials. This high-energy radiation is particularly advantageous for uncovering the intricate details of surface reconstruction processes, making it a pivotal tool in future research.
(5) The discrepancy between laboratory test conditions and industrial electrolysis environments poses a significant challenge in accurately capturing the surface reconstruction behavior of electrocatalysts. Understanding these processes under realistic operating conditions is imperative for bridging the gap between fundamental research and industrial applications. The development of in situ characterization technologies capable of replicating industrial conditions and capturing real-time surface reconstruction dynamics will be instrumental in advancing the field.

## Data Availability

No new data were created or analyzed in this study. Data sharing is not applicable to this article.
